# A Comparison of End-to-End Decision Forest Inference Pipelines

**DOI:** 10.1145/3620678.3624656

**Published:** 2023-10-31

**Authors:** Hong Guan, Saif Masood, Mahidhar Dwarampudi, Venkatesh Gunda, Hong Min, Lei Yu, Soham Nag, Jia Zou

**Affiliations:** 1 Arizona State University; 2 IBM T. J. Watson Research Center; 3 Rensselaer Polytechnic Institute

**Keywords:** Machine Learning System, Decision Forest

## Abstract

Decision forest, including RandomForest, XGBoost, and LightGBM, dominates the machine learning tasks over tabular data. Recently, several frameworks were developed for decision forest inference, such as ONNX, TreeLite from Amazon, TensorFlow Decision Forest from Google, HummingBird from Microsoft, Nvidia FIL, and lleaves. While these frameworks are fully optimized for inference computations, they are all *decoupled* with databases and general data management frameworks, which leads to cross-system performance overheads. We first provided a DICT model to understand the performance gaps between decoupled and in-database inference. We further identified that for in-database inference, in addition to the popular UDF-centric representation that encapsulates the ML into one User Defined Function (UDF), there also exists a relation-centric representation that breaks down the decision forest inference into several fine-grained SQL operations. The relation-centric representation can achieve significantly better performance for large models. We optimized both implementations and conducted a comprehensive benchmark to compare these two implementations to the aforementioned decoupled inference pipelines and existing in-database inference pipelines such as SparkSQL and PostgresML. The evaluation results validated the DICT model and demonstrated the superior performance of our in-database inference design compared to the baselines.

## 1 INTRODUCTION

Decision forest models, such as RandomForest [20], XGBoost [25], and LightGBM [36] are widely used machine learning algorithms for classification and regression tasks over tabular data. They are widely applied in production scenarios, including credit-card fraud prediction, recommendation, business intelligence, etc, over tabular data such as transaction records and customer profiles. According to Kaggle user study [11], 78.1% of data scientists use decision trees or random forest, and 61.4% use XGBoost and LightGBM, while the usages of convolutional neural networks, fully connected neural networks, and transformer networks are only 43.2%, 28.2%, and 14.8% respectively. Compared to deep neural network models that are considered opaque, decision forest models are easier to identify and explain the significant variables in the data [15], which is important when applied to business decision processes that have compliance and audit requirements. They are popular for their robustness, scalability, and their abilities to handle a large number of features, handle missing data, and work well with both linear and non-linear relationships [18, 24, 28, 34, 45].

Given the importance of decision forest models, there are many popular open-source systems recently engineered to support and optimize the inference process of decision forest, including Scikit-Learn [44], ONNX [17], TensorFlow Decision Forest (TFDF) [31], TreeLite [26], HummingBird [41], lleaves [4], and Nvidia FIL [9, 10]. However, we observed several shortcomings in these systems, as explained below.

### 1. The data management gap.

All of these systems decouple the inference computation and data management and thus introduce additional latency for loading data from/to external data stores. While popular database services such as Amazon Redshift [42], SQLServer [29, 43], Google Big-Query [30], PostgresML [7] all support integrating a model predict function into the SQL query interface, under the hood, they all choose to delegate the inference computations to external ML runtimes such as SageMaker [37], ONNX [17], TensorFlow [14], and XGBoost [25]. Although recent works such as Raven [43] proposed to co-optimize the SQL processing and ML processing data-driven model pruning and model-based feature pruning, significant portions of ML computations still need to run in the ONNX runtime, which is expensive [43].

### 2. The in-database (in-DB) inference performance gap.

Existing database systems, such as SparkSQL [16], also support native and fully integrated decision forest inference processing, by encapsulating the prediction logic into a User Defined Function (UDF). However, we found such implementation may lead to sub-optimal performance compared to the aforementioned popular ML frameworks. First, we observed a significant amount of cache misses while encapsulating a relatively large model into UDFs. Second, the query parsing, optimization, and compilation overheads are repeated for processing different inference queries involving the same model. Third, there are additional overheads associated with relational processing, e.g., catalogs, caching, thread synchronization brought by bulk synchronous parallelism, etc. Such a performance gap limits the performance gain that can be achieved by the in-database implementation, compared to popular ML frameworks.

### 3. The end-to-end performance understanding gap.

Recently, there are emerging works designing new systems to support ML from SQL, such as Tensor Relational Algebra [32, 33, 51], Daphne IR [27], LingoDB [35], etc. However, insufficient works explain how (de)coupling ML frameworks and data management frameworks may impact the end-to-end latency of inference workloads, which are critical for choosing underlying system designs.

To close the gaps, we face key challenges including intricate relationships between end-to-end latency and factors like inference representation, model complexity, and dataset traits; diverse design choices for in-database decision-forest inference; and varied decoupled inference platforms. Our key contributions include:

#### 1. DICT performance modeling. (Sec. 3)

To close the end-to-end performance understanding gap, we established a simple yet powerful DICT model for understanding when in-database (in-DB) inference should be used. The model highlighted two fundamental guidelines for this work: (1) In-DB inference would be desirable if the data transfer becomes the bottleneck. (2) Closing the inference performance gap between ML systems and DB systems is important to make in-DB inference attractive.

#### 2. A high-performance in-DB decision forest inference system with multiple representations. (Sec. 4)

We designed and optimized a prototype of an in-DB decision forest inference system, on top of netsDB, which is an object-oriented relational database developed in C/C++ [32, 33, 47, 51, 53–58].

We implemented different representations of the decision forest inference in RDBMS. The popular **UDF-centric** representation encapsulates the entire inference computation into a single UDF. However, we found that when the size of the model increases, the cache locality worsens quickly using this approach. The **relation-centric** representation breaks down the inference computation into a flow of relational operators, including a cross-product operator that pairs up each decision tree and a block of testing samples. Then, for each pair, the prediction function from the decision tree is invoked to predict for each sample in the block. This operator is followed by an aggregate operator that aggregates all partial prediction results. We implemented both representations using batching and vectorization. In addition, the UDF-centric representation adopts data parallelism so that each thread invokes the UDF on a data partition. The relation-centric representation adopts model parallelism, e.g., the cross-product operator is launched in multiple threads, with each thread responsible for a model partition (i.e., a subset of decision trees). The relation-centric representation requires some model processing job stages. The output of these job stages can be reused for running inferences of the same model on different testing datasets. We thus proposed the model-materialization technique so that the output of such job stages is materialized to accelerate executing different inference queries that involve the same model.

#### 3. A comprehensive benchmark study and detailed performance analysis. (Sec. 5)

We compared our optimized in-database inference framework on netsDB, existing in-database inference frameworks such as SparkSQL, and the aforementioned *decoupled* decision forest inference frameworks including Scikit Learn/XGBoost/LightGBM, HummingBird, TreeLite, lleaves, ONNX, NvidiaFIL, on a broad class of workloads at all scales using RandomForest, XGBoost, and LightGBM with different levels of model complexity. The types of datasets range from sparse to dense and narrow to wide. We compared the end-to-end latency of the decision forest inference process, including data loading, inference, and result writing. The evaluation results not only validated the DICT model but also showed the performance superiority of our in-database inference framework: It achieved up to more than **100**× speedup compared to the best of the decoupled pipelines, up to **22**× speedup compared to in-database baseline such as SparkSQL, among other surprising observations. The benchmarking framework is fully automated and open-sourced (https://github.com/asu-cactus/DFInferBench).

## 2 BACKGROUND

RandomForest [20], XGBoost [25], and LightGBM [36] are three different decision forest training algorithms. Even training on the same dataset, with the same number of trees and the same maximum depth of each tree, the trained models could have different shapes using these training algorithms [41]. The inference processes of the three decision forest models share the same first phase, which is to obtain the exit leaf for each tree in the forest. The second phase is slightly different. Taking Scikit-learn implementations of binary classification as an example, in the second phase, the RandomForest algorithm averages all trees’ exit labels and then applies a sigmoid function to convert the averaged value to a probability score. For XGBoost and LightGBM, the second phase sums up the weights of all exit labels and then obtains the final prediction using a sigmoid function. In this section, we will first introduce the baseline platforms that are evaluated in our comprehensive benchmark study. Then, we describe and justify the targeting benchmark scenarios.

### 2.1 Decision Forest Platforms

We describe the design principles of popular decision forest platforms, which are used as the baselines for our evaluations, from three aspects: inference algorithm, parallelism, and vectorization.

#### Scikit-learn [44]

**Figure 1:**
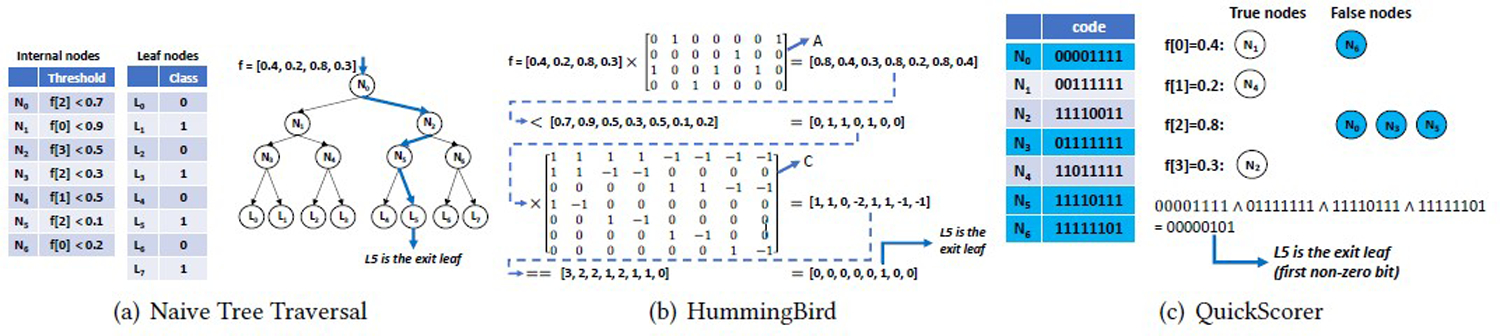
Illustration of Decision Forest Inference Algorithms.

It implements its own RandomForest algorithm but invokes the XGBoost [25] and LightGBM [36] libraries to train and make inferences corresponding types of models. They all implement the naive tree traversal algorithm for inference, as illustrated in Fig. 1(a). Scikit-learn used model parallelism for random forest prediction; each thread runs the inferences of a partition of trees over the input data, and the results will be used to update a shared result vector protected by a lock. The predict function is vectorized by taking a batch of samples as input. Both XGBoost and LightGBM libraries adopt data parallelism. The XGBoost library also uses vectorization, while the LightGBM does not.

#### ONNX [17]

It also uses the naive tree traversal algorithm. It chooses data parallelism or model parallelism based on the number of input samples and the number of trees in the forest. It does not exploit vectorization, and the tree traversal function takes a single sample as input at a time.

#### HummingBird [41]

It transforms the tree traversal process into tensor computations, as illustrated in Fig. 1(b). It first converts the decision tree structure to two main tensors: (1) A tensor A that represents the relationships between each internal node and each feature; (2) A tensor C that captures the parent-child relationships among each pair of internal nodes. Then, the tensor of input samples is multiplied with tensor A to obtain the input path tensor. After that, the input path tensor is multiplied with tensor C to obtain the output path tensor. This way, existing tensor libraries on the CPU and GPU can be leveraged to accelerate the prediction process.

#### TreeLite [26]

It imports external models and partitions the trees into several compilation units. These compilation units will be transformed into C source functions in parallel. Each C source function corresponds to a compilation unit (i.e., a shared library file). It takes a single sample as input, runs a series of nested if-else blocks, and outputs the final predictions for trees belonging to the compilation unit.

#### Nvidia FIL [9, 10]

Each GPU thread is responsible for inferring a batch of samples on one tree. To optimize the GPU cache locality, it exploits a reorganized dense tree representation in GPU memory, where the nodes at the same level but from different trees will be stored together to improve the cache locality. It also implements a sparse tree storage format, where the nodes from all trees are stored in one flat array. While nodes from one tree are stored together, sibling nodes that share the same parent node are always stored adjacently. To reduce branch misses, it exploits a predicate to replace the conditional branch. The conditional branch: **if** (cond) **return** left(node_idx) **else return** right(node_idx), is replaced by **return** left(node_idx) + cond, which avoids branches.

#### lleaves [4]

It also compiles trees to nested if-else blocks. However, instead of translating the model into C source codes for compilation, lleaves designs an intermediate representation to describe the models and leverages the LLVM framework for code generation. Notably, lleaves is more optimized than TreeLite. For example, lleaves can support model parallelism by chunking the model, and the functions generated by lleaves support vectorization. Lleaves currently only supports the LightGBM model on the CPU.

#### Apache SparkSQL [16, 40]

SparkSQL invokes MLlib APIs to run decision forest inferences over data frames using a UDF-centric representation. The implementation of the model is based on the naive tree traversal algorithm. Under the hood, SparkSQL applications will be lazily optimized by the Catalyst query optimization. The implementation takes one sample as input at each time, and it relies on code generation to alleviate virtual function call overheads [16].

#### Additional Notes.

We actually investigated two more systems: **1. TensorFlow Decision Forest (TFDF)** [12] It wraps a C++-based Yggdrasil library [31], which implements the QuickScorer algorithm [38, 39, 50] as well as the naive tree traversal algorithm. It will benchmark and select the best algorithm at the model compilation stage. The QuickScorer algorithm is illustrated in Fig. 1(c). It converts the tree traversal into bit-wise AND operations. However, at this point, only single-threaded is supported for the inference process. so we only included TFDF in a single-thread benchmark (Tab. 10). **2. PostgresML** [7] supports decision forest models using UDF-centric representation. Under the hood, the inference computation invokes the XGBoost/LightGBM API. However, it cannot import our pre-trained model; the model trained using their system is also opaque to the users. We used PostgresML to train XGBoost models on Higgs and Fraud (see Tab. 1) using the same hyper-parameters and observed similar accuracy with our pre-trained XGBoost model on these datasets. Then, we compared the inference latency using the high-performance predict_batch API in PostgresML. The results showed that netsDB significantly outperformed PostgresML on Higgs, achieving 9×, 2×, and 2× speedup for 10-tree, 500-tree, and 1600-tree models, while their performance on Fraud, which is a small dataset, is similar. The training accuracy on other datasets or using LightGBM is very different from our pre-trained models, so we omitted the comparison in this work.

### 2.2 Targeting Scenarios of This Work

There are two end-to-end inference scenarios: (1) Features are extracted from the tables, and then inferences are computed over the features. (2) Features are preloaded into a relation. In this work, we focus on the second scenario, because of the following reasons:

First, in data science pipelines, the prepared features are often materialized first and then scored using multiple models for different purposes [21]. For example, given a customer’s transaction, people use different models for product recommendation, churn prediction, lifetime value prediction, etc. Given patient features, people use different models to predict the probability and severity of each disease.

**Figure 2:**
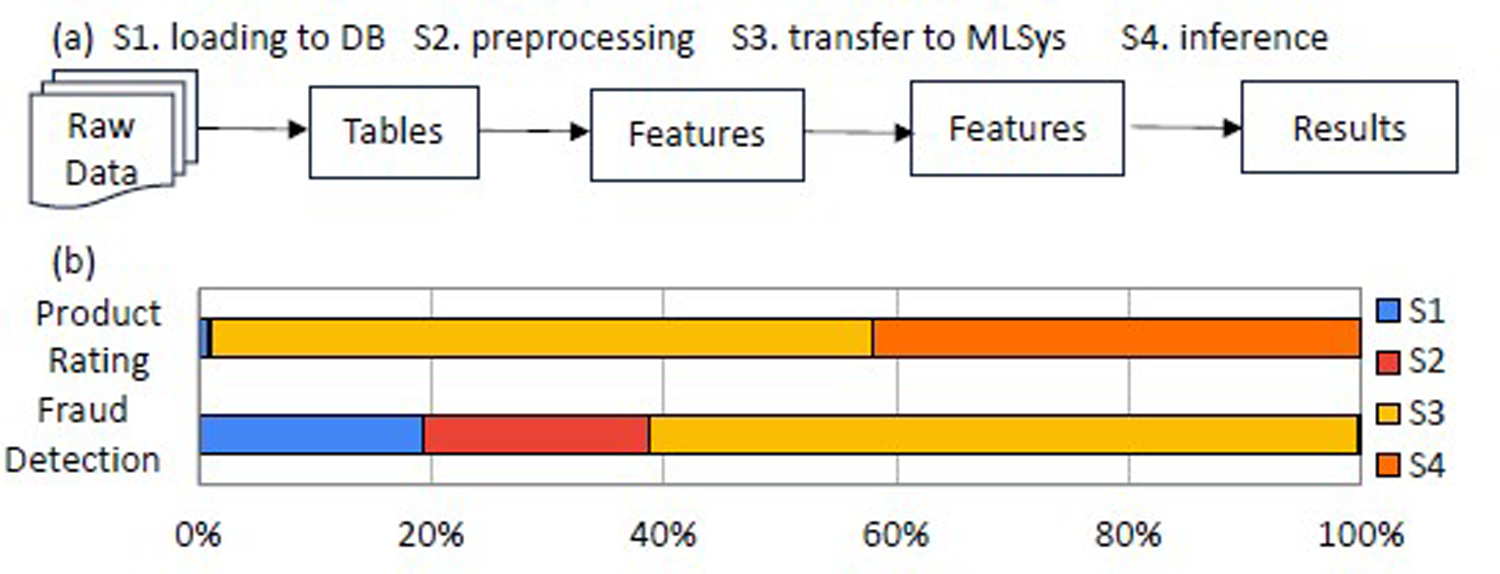
(a) Decoupled inference pipeline; (b) End-to-end latency breakdown for two TPCx-AI use cases using SF=30.

Second, the feature exaction is not the performance bottleneck for a significant portion of workloads. As illustrated in Fig. 2, we analyzed the breakdown of the end-to-end latency for two representative in-database ML workloads using TPCx-AI reference code. (The Product Rating workload uses SVD for inference, and the dataset has 208, 940 samples. The Fraud Detection workload uses logistic regression for inference, and its data size has 14, 477, 835 samples, and thus significantly more time is spent in data processing.)

Third, for in-database inference, it is possible to leverage the physical database design optimizer to store the features as a collection of tensor blocks to accelerate the inference workloads that will consume the features.

Importantly, in all of our experiments, we targeted **batched inference** scenarios, because it is much more popular than online inference in production, based on the analysis of **130** Microsoft customer engagement [43].

#### Other Related Works.

In netsDB relation-centric representation, the model materialization idea shares some similarities to view materialization [19]. The cross-product operator is also used in many other scenarios, such as joining a data stream and a query stream [22, 48]. However, the integration of these techniques for serving decision forest workloads and related evaluations is novel.

## 3 THE DICT PERFORMANCE MODELING

In order to understand the end-to-end inference performance of the dedicated machine learning platform and the in-database machine learning, we developed a model based on the following parameters:

D: the dataset’s size (in bytes) to be inferred.

I: the inference throughput (i.e., the maximum number of tuples that can be inferred per second) for the target inference platform. I is highly related to system engineering factors, and the model complexity. Particularly, an inverse proportion exists between the inference throughput I and the model algorithm complexity.

C: the cardinality (i.e., number of tuples) of the dataset.

T: the data transfer throughput (i.e., the maximum number of bytes that can be transferred per second) between the data source and the target inference platform.

**Figure 3:**
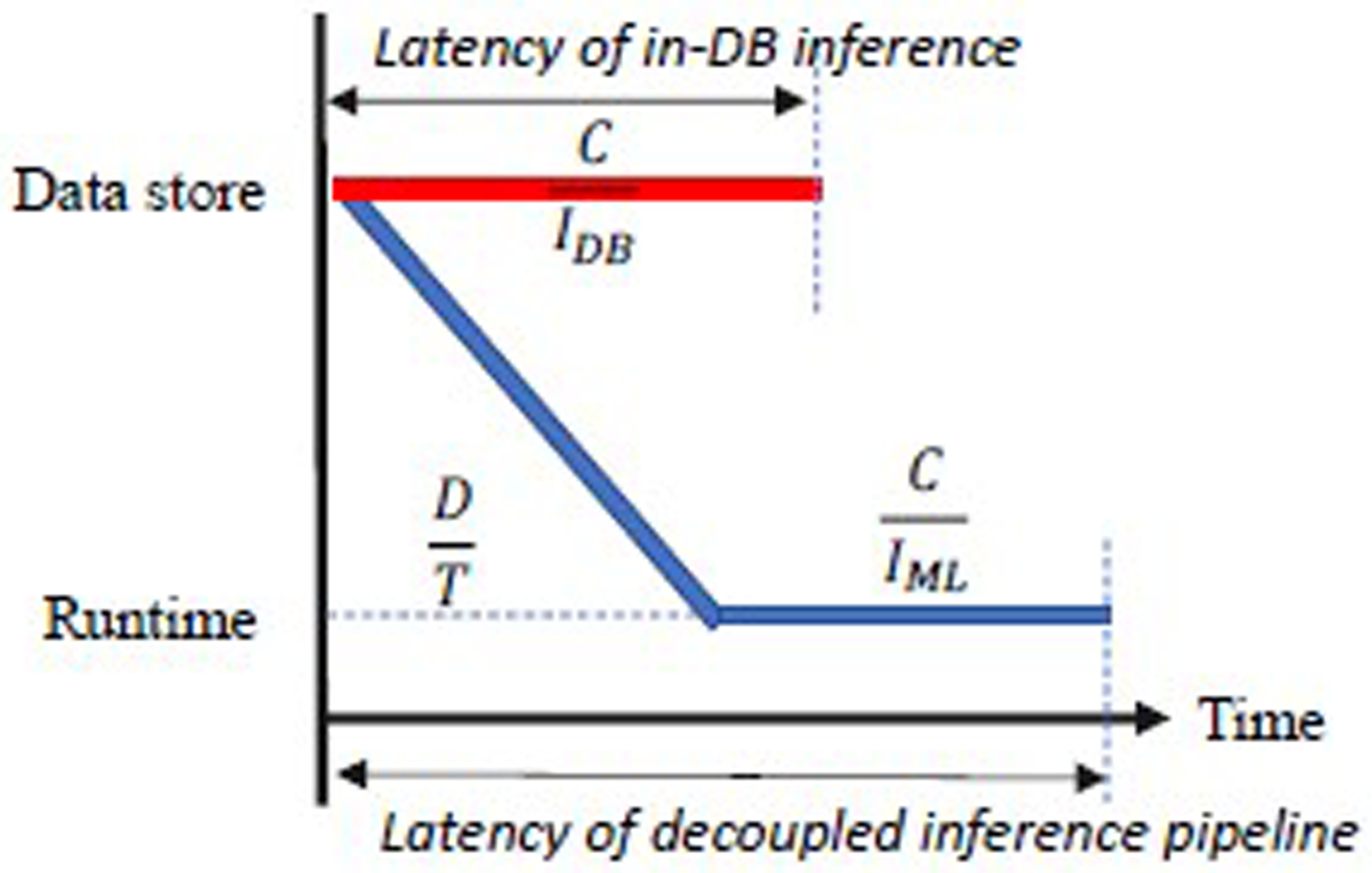
The DICT model

We further estimate the speedup of the in-database inference compared to the decoupled inference pipeline as $\frac{\frac{D}{T}+\frac{C}{I_{ML}}}{\frac{C}{I_{DB}}}$, as illustrated in Fig. 3. The **key conclusions** include:

(C1) When fixing $\frac{I_{DB}}{I_{ML}}$, the higher of the transferring overhead (i.e $\frac{D}{T}$), the better speedup for the in-database inference.

(C2) When fixing D and T (i.e., the transferring overhead), increasing $\frac{I_{DB}}{I_{ML}}$ will increase the speedup brought by the in-database inference.

(C3) Wide and short datasets gain more from in-database inference than narrow and tall ones. That’s because when fixing T and $\frac{I_{DB}}{I_{ML}}$, the larger number of features, as correlated to $\frac{D}{C}$, the better speedup for in-database inference.

(C4) When fixing D, C, and $\frac{I_{DB}}{I_{ML}}$, the smaller of the data transfer throughput T, the better speedup for in-database inference.

(C5) When fixing C, and T, and the ratio of $\frac{I_{DB}}{I_{ML}}$, the larger of the value of $D\times I_{DB}$, the better speedup for in-database inference. As mentioned, I decreases with the increase in model complexity, denoted as M. Therefore, fixing other factors, the larger value of $\frac{D}{M}$, the better speedup for in-database inference.

While this paper focuses on the inference of decision forest models, these conclusions generalize to other AI/ML models.

## 4 OUR IN-DATABASE INFERENCE DESIGN

In this section, we will explore the optimal implementation for decision forest inference workloads in RDBMS to close the aforementioned inference performance gap.

We found that the naive tree traversal algorithm is best suited for RDBMS. The HummingBird algorithm relies on expensive matrix computations, and it is not suitable for most RDBMSs that do not support GPU acceleration. The QuickScorer algorithm is hard to be efficiently represented in a relation-centric style. One reason is that the QuickScorer model groups all nodes by features; thus, the model is essentially a collection of feature groups. Because the sizes of feature groups are not well-balanced, it is challenging to partition the model evenly. In addition, as demonstrated in our benchmark, the most competitive runtimes such as ONNX, Scikit-Learn/XGBoost, and Nvidia FIL are all based on the naive tree traversal algorithm. We thus chose to use the naive tree traversal algorithm to implement various in-DB representations of decision forest inferences.

These representations are implemented in netsDB, which is an open-sourced object-oriented relational database built using C/C++ [32, 33, 47, 51, 53–58] for fair comparison to baselines, most of which are also implemented in or backed by C/C++. But they can be implemented in any database system that supports UDFs.

### 4.1 The Underlying RDBMS

Similar to other dataflow frameworks such as Spark/SparkSQL [16, 52], on netsDB, the users develop their applications as dataflow graphs, where every node represents a relational operator (which should be customized using UDFs) and every edge represents a dataset. In netsDB, a dataset is abstracted as a collection of objects, which is similar to a Resilient Distributed Dataset (RDD) in Spark.

A dataflow graph is split into multiple pipeline (job) stages at runtime. A pipeline stage is a series of atomic operators (e.g., scan, transform, hash, partition, probe, join, cross-product, aggregate, write) with the last operator being a pipeline breaker that materializes the output data (e.g., hash, partition, aggregate, write). For example, a join or aggregate relational operator will be split into multiple atomic operators belonging to different pipeline stages. Each operator is fully vectorized and takes a list of tuples as input at each invocation. Operators within the same job stage are pipelined (i.e., fused). Because each relational operator is visible to the users and the atomic operators that are lowered from the relational operators are all developed in templated classes, code will be generated through C++ templated meta-programming.

Each pipeline stage is executed in multiple threads. Each thread executes a loop. At each step, the thread fetches a page of the input dataset from the buffer pool, processes the page, and writes the outputs to an output page, which is also cached in the buffer pool. The buffer pool is governed by the Least Recently Used (LRU) policy.

### 4.2. System Overview

While loading a model, the system generates UDF-centric (Sec. 4.3.1) or Relation-centric representations (Sec. 4.3.2), or use a rule-based model to select the representation automatically (Sec. 4.3.3). The predict() function of the model will be lowered to execute a dataflow graph corresponding to UDF-centric or relation-centric representations respectively. We identify that when the dataflow graph involves multiple pipeline stages (e.g., as in relation-centric representations), some stages will be repeatedly executed across different queries (e.g., for running the model on different inference datasets). We thus optimize the graph by materializing and reusing the results of the pipeline stages (Sec. 4.4). In order to reduce the overheads of virtual function calls, and make use of SIMD instructions, the input samples are stored as a collection of tensor blocks, called sample blocks. Each block is a 2D tensor that represents a vector of samples. Physically, the collection of tensor blocks, termed as a *data set*, is stored as a collection of pages containing one or more tensor blocks. The pages are cached in the buffer pool, governed by the least recently used (LRU) page replacement policy.

### 4.3 Model Representations

#### 4.3.1 UDF-Centric Model Representation.

With the UDF-centric representation, the decision forest inference logic is encapsulated in a single UDF that customizes a transform operator (i.e., like a map function). The UDF contains a forest object, which is a vector of binary trees, and each tree is stored as a vector of ordered tree nodes. The UDF has a prediction function that takes a sample block as input and outputs a block of predictions. The prediction function iteratively processes each sample in the block by traversing each tree and aggregating the prediction of all trees.

The dataflow graph for a simple example of UDF-centric inference consists of a transform operator that is customized by a UDF, which takes a sample block as input and outputs a block of inference results. After compilation, the dataflow graph is scheduled as one pipeline stage. The pipeline stage is executed by multiple threads (corresponding to the total number of CPU cores) in a data-parallel style. Each thread iteratively fetches a vector of sample blocks, runs the prediction UDF over it, and writes the final predictions to an output dataset.

The benefit of the UDF-centric approach is in its simplicity. For example, the inference process is compiled into a single pipeline stage. The encapsulation also facilitates extending the UDF to invoke functions from popular libraries, including GPU libraries (with a potential challenge in coordinating the database thread scheduling and the UDF runtime scheduling). One shortcoming is that each worker thread needs to access the whole forest model for running inferences on a data partition, which leads to significant cache misses for large-scale models. We address the issue in Sec. 4.3.2.

#### 4.3.2 Relation-Centric Model Representation.

The decision forest model can also be represented as a collection of trees, stored in a *model set*. The inference process is then compiled into a dataflow graph, which uses two key operators: (1) A cross-product operator, which performs a Cartesian product between the collection of trees and input sample blocks, enumerates all possible pairs of tree and sample blocks. Each pair is further converted into a block of prediction results (e.g., the return class or weight associated with the exit leaf) using a fine-grained transform UDF that invokes each tree’s predict() method over each block. (2) An aggregate operator is used to aggregate all prediction results for the same sample to derive the final result. The aggregation logic for RandomForest, XGBoost, and LightGBM are slightly different and are encapsulated into fine-grained UDFs. Note that although this representation is termed as *relation-centric*, UDFs are used to customize the operators such as transform and aggregate, except that compared to the UDF-centric approach, the granularity of the UDFs in the *relation-centric* representation is much smaller.

To reduce cache misses, we implemented a cross-product operator in a model-parallel fashion. It partitions the model into many pages, and each page has a subset of decision trees. The pages will be dispatched to threads. Using this approach, each thread only needs to access a partition of trees at a time. As observed in our experiments, it significantly reduces the cache misses and shortens the inference latency for large-scale models.

However, we also found that this approach is sub-optimal for small-scale models. That is because compared to the UDF-centric representation that only requires one pipeline stage, the relation-centric representation is compiled to multiple pipeline stages because multiple pipeline breakers exist in the dataflow graph. For example, one stage partitions the model, one stage runs the cross-product and groups partial prediction results by block IDs, one stage aggregates the prediction results from all trees, and one stage post-processes the aggregated result (e.g., applying the sigmoid function) and writes the final output to a dataset. Each pipeline stage introduces data scanning and materialization overheads. In addition, because each stage is running with multiple threads, it also involves synchronization overheads.

Though in this work, UDF-Centric and Relation-Centric decision forest inference is implemented on netsDB, they can generalize to a large number of database systems, e.g., those that use exchange operators and pipelining (fusion) of operators and those that follow the MapReduce framework [47].

#### 4.3.3 Dynamic Representation.

We designed a dynamic representation, which relies on a set of rules to choose the UDF-centric or the relation-centric representation when loading the model. In current practice, we learn the rules by training a single decision tree classification model over a trace of 1000 inference executions from decision forest models of a varying number of trees and varying tree depths over our benchmark datasets (Sec. 5). The model considers features such as tree number, tree depth, cardinality of the dataset to be inferred, and number of features in the dataset. The model outputs 1 if the relation-centric representation is chosen, and 0 if the UDF-centric representation is chosen. Our current work implements in-DB inference primarily on an AWS r4.2xlarge CPU platform, but this dynamic representation can be easily extended to various hardware configurations by including those configurations as features of the decision tree classifier.

### 4.4 Model Materialization and Stage Reuse

To further optimize the relation-centric representation, we proposed the model materialization strategy. We identified that the model-partitioning stage can be reused by different inference applications as long as they use the same model. Therefore, we can materialize the results of this pipeline stage and directly reuse the materialized results to simplify the dataflow graph.

To implement the idea in netsDB, we modified the query optimization process to recognize such a reusable model set and generate the pipeline stage that partitions the set and prepares it for the cross-product operation. This pipeline stage will be executed, and its results will be stored in a *materialized set*. When there is an inference query, the materialized set will be directly used, and the execution of the corresponding pipeline stage will be skipped.

In addition, we observed that: (1) For handling inference queries involving relatively small datasets and models, a significant portion of the time is spent optimizing the dataflow graph and generating pipeline stages. (2) For different queries that follow the UDF-centric representation and use the same model, the pipeline stages are similar to each other, except the input data set is different. (3) For different queries that follow the relation-centric representation, even using different models, the pipeline stages are similar to each other, except the input data set and model set are different.

Therefore, we materialized the pipeline stages and shared and reused these stages across the execution of different inference queries to reduce the query processing time.

## 5 A BENCHMARK STUDY

### 5.1 Benchmark Workload Description

We evaluated the performance of various decision forest models at all scales on well-known classification and regression datasets, as shown in Tab. 1. Most of them are widely used in various batch inference benchmarks such as GBM-perf [2], LightGBM benchmark suite [5], Nvidia gbm-bench [6], Hummingbird benchmark [41], and so on. They represent diverse datasets that vary in domains, sizes, number of features, sparsity, and density. The prediction type, i.e., classification or regression, does not affect the conclusions because the inference computation of regression and classification models are very similar. Based on top of those benchmarks, we added the fraud detection use case from TPCx-AI [13] and the Criteo [1] classification to our benchmark study. TPCx-AI is a well-known in-database AI benchmark, and Criteo is widely used in ML benchmarks [46].

In this work, we use Scikit-learn to train models using RandomForest, XGBoost, and LightGBM algorithms on each evaluation dataset in Tab. 1. For each type of model, we used 10, 500, and 1, 600 trees, with each tree having a maximum depth of 8. These are all widely used hyper-parameters [26, 41]. We then convert these models to be loaded to each platform for inferences. The performance of the model conversion and loading process has been discussed in Sec. 5.7.

For TPCx-AI, we trained the model on a smaller dataset with scale factor (SF) 1, then tested the model on the dataset with SF=30, which is described in Tab. 1. For Criteo, the training and testing data was pre-split by the data provider [1, 3], as illustrated in Tab. 1. For all other workloads, we used 80% of samples to train models. Then we convert the trained model for each target platform and run inferences against the 20% remaining samples.

#### Benchmark Scenarios.

In this work, we focused on comparing the in-DB inference approach and the decoupled inference approach. For in-database inference approach such as netsDB and SparkSQL, the testing datasets were stored natively. In SparkSQL, we persisted the dataframe in memory, and only measured the inference time. For other platforms following the decoupled approach, the testing datasets, except for Epsilon and Criteo, were stored in tabular format in a PostgreSQL database installed on the same machine, with the database connection accelerated using the state-of-art ConnectorX library [49]. Besides, Epsilon has 2000 features, and Criteo has 1 million features, but PostgreSQL only supports up to 1600 columns [8]. Therefore, we stored each tuple in Array type in PostgreSQL for Epsilon, which turned out to be slower than the tabular format in data loading as detailed in Sec. 5.5.1. For Criteo, we loaded it from a LIBSVM file in sparse storage format [23].

We measured data loading time, inferences time, and result writing time in the end-to-end inference pipeline. We did not consider the model conversion and model loading time because these times can be amortized to multiple inference queries. We will discuss such one-time costs in Sec. 5.7. We did not consider the throughput metrics, e.g., how many inference queries or how many tuples are handled per second, because the former is irrelevant to the batch-serving scenario, and the latter can be directly obtained as the inverse of the latency.

### 5.2 Experimental Configuration

#### Software Configuration.

We used Scikit-learn v1.1.2, ONNX v1.12.0, Hummingbird v0.4.5, Nvidia Rapids-FIL v22.08, TreeLite v2.3.0, TFDF v0.2.7, PostgreSQL v14, Apache SparkSQL v3.2. For lleaves, and ConnectorX, we used the code downloaded from their Github master repositories. For all platforms, we carefully tune the number of threads, batch size, block size, as well as other parameters to fully optimize the performance. We ran the HummingBird models in several different backends, including Pytorch v1.13.1, TorchScript (within Pytorch v1.13.1), and TVM v0.10.0, and select the best results for with and without GPU acceleration.

#### Hardware Configuration.

For CPU experiments, we used the AWS EC2 r4.2xlarge instance with 8 CPU cores and 62 gigabytes of memory. All instances are installed with Linux Ubuntu 20 and 200 gigabytes SSD storage. The cost of the instance is $0.532 per hour (which we termed as **CPUCost**). For the GPU experiments, we used an AWS g4dn.2xlarge instance, which has an NVIDIA T4 Tensor Core GPU with 16 gigabytes memory and an eight-core CPU with 32 gigabytes host memory. Its cost is $0.752 per hour (which we termed as **GPUCost**). We will investigate more hardware types in the future. To handle the performance variations in cloud environments, we repeated each experiment multiple times. In total, we used more than ***10,000*** hours on AWS EC2 for the evaluation.

We used Linux Perf to profile the elapsed time as well as cache misses. We used gpustat, a wrapper built on nvidia-smi, for profiling the GPU utilization and memory utilization.

### 5.3 Results on Small-Scale Dense Datasets

In this section, we will summarize the results on two relatively smaller datasets: Fraud and Year, as described in Tab. 1.

**Figure 4:**
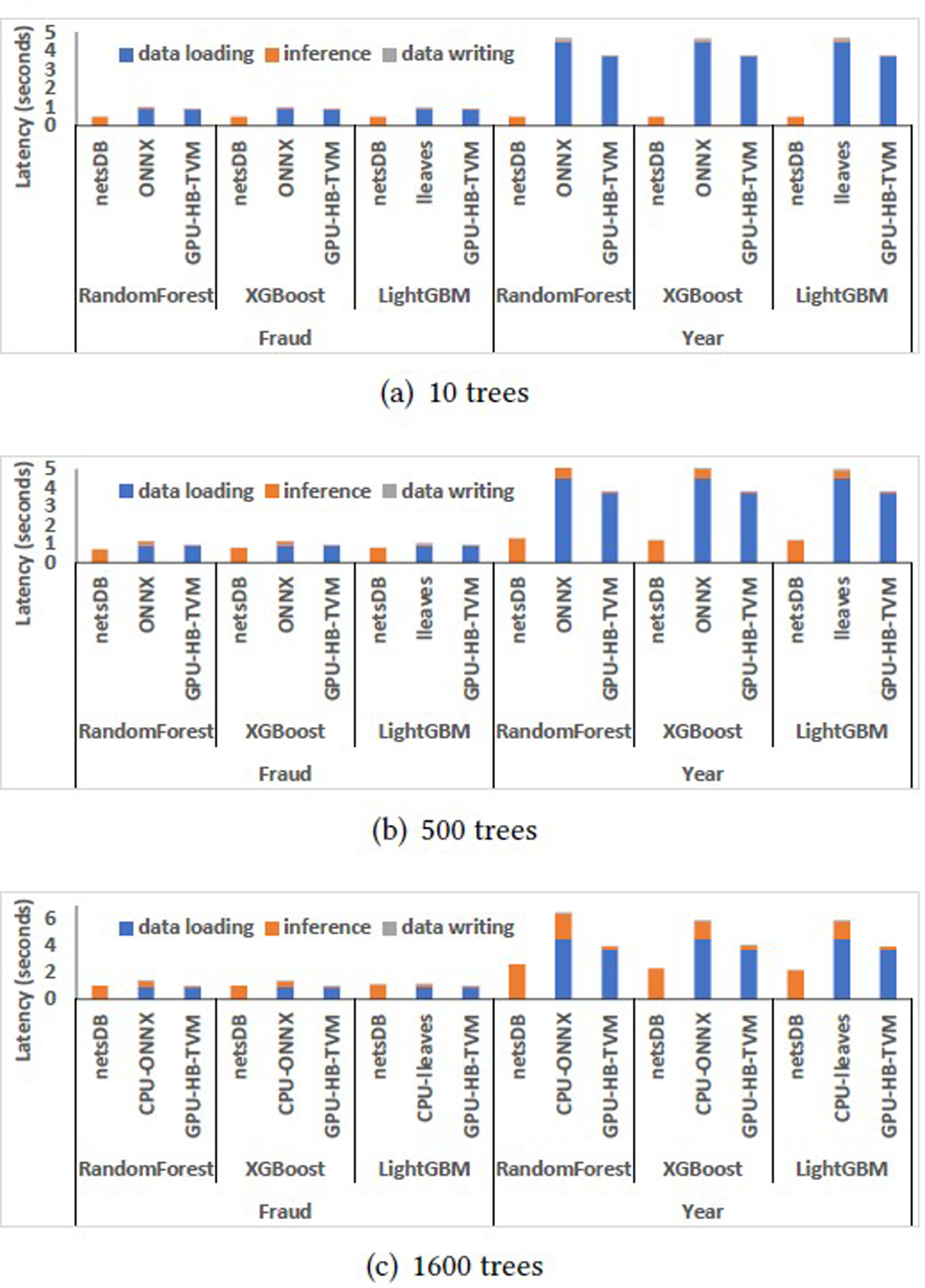
Latency breakdown for netsDB, the fastest of the rest CPU platforms, and the fastest GPU platform on small datasets.

As illustrated in Tab. 2 and Tab. 3, among all CPU/GPU platforms, the UDF-centric representation in netsDB (db-UDF) achieved the lowest latency for small models that have 10 trees. netsDB with the model materialization and stage reuse optimization (db-OPT) significantly reduced the latency for the relation-centric representation (db-rel) and achieved the lowest latency for 500 and 1600 trees across all platforms. That is because inference on such small datasets is significantly faster than data transfer, and data transfer thus becomes the major bottleneck, as illustrated in Fig. 4. This bottleneck is alleviated using the in-DB inference.

#### 5.3.1 Fraud.

We have the following key observations on this workload. First, netsDB outperformed other CPU/GPU platforms. That is because data transfer is the major bottleneck of the Fraud workload, as illustrated in Fig. 4. When applying 10-tree models to the Fraud dataset, data transfer accounts for 90% of overall latency on ONNXCPU, 95% on lleaves, and 97% on HB-TVM. However, the ratio of data transfer latency to the overall latency decreases with the increase in model sizes. For example, for 1600-tree models, the aforementioned ratios dropped to 65%, 76%, and 88% respectively. Second, db-rel performed significantly worse than other CPU/GPU platforms for models with 500 and 1, 600 trees. That is mainly because it runs multiple pipeline stages, including partitioning the model to prepare for the cross-product operation. The scheduling and materialization overheads are significant compared to the inference latency. The model materialization and stage reuse technique, as used in db-OPT, resolved the issue and improved the performance, as illustrated in Tab. 2. Third, netsDB, ONNX, and lleaves achieved better monetary costs than GPU platforms (see Sec. 5.2 for AWS cost information). It indicates that GPU may not be very helpful for the inferences on small datasets (e.g., small samples are batched in small-size buffers to guarantee low buffering latency).

#### 5.3.2 Year.

The observations for the Year workload are similar to Fraud, except that netsDB achieved significantly higher speedups on the Year dataset. For example, as illustrated in Tab. 3, for small models with 10 trees, db-UDF achieved 7.6× speedup compared to the best GPU platform (10.8× speedup after normalizing the GPU latency by cost), and 9.4× speedup compared to the best of the rest CPU platforms. But the corresponding speedups achieved on the Fraud dataset were merely 1.8× and 2×, respectively. Then, for 500-tree models, db-OPT achieved 3× speedup (4.5× normalized speedup) compared to the best GPU platform and 4× speedup compared to the second-best CPU platforms, while the corresponding speedups achieved on the Fraud dataset was merely 1.1 to 1.3× for CPU and 1.1 to 1.5× for GPU. In addition, for the case of 1, 600-tree models, for the Year dataset, db-OPT achieved 1.7 to 2.5× speedup (2.2 to 2.6× normalized speedup) compared to the best GPU platform and 1.5 to 2.7× speedup compared to the second-best CPU platforms. However, for the same case, there was no significant speedup achieved on the Fraud dataset by db-OPT compared to GPU platforms, and the second-best CPU platforms.

As illustrated in Fig. 4, the data loading and writing times accounted for 96%, 96%, and 98% on ONNXCPU, lleaves, and HB-TVM, respectively, which were even higher than the corresponding ratios for the Fraud workload. This explains the better speedup achieved on Year. Similar to Fraud, the ratio of data loading latency to the overall end-to-end latency decreased with the increase in model sizes. For 1600-tree models, the aforementioned ratios dropped to 69%, 77%, and 94% for the Year case.

### 5.4 Medium to Large-Scale Dense Datasets

This section mainly investigates the performance of three workloads: Higgs, Airline, and TPCx-AI. The overall benchmark results for these datasets are illustrated in Tab. 4, Tab. 5, and Tab. 6. When processing those larger-scale datasets, the performance gain of db-OPT is small, because the inference computation, rather than model preparation, becomes the performance bottleneck.

#### 5.4.1 Higgs.

As illustrated in Tab. 4, when only using CPU, for a 10-trees model, db-UDF achieved 8 to 10 times speedup compared to the fastest of the rest CPU platforms. When using 500 trees, db-rel achieved 1.3× to 1.6× speedups compared to the fastest of the rest CPU platforms for RandomForest, XGBoost, and LightGBM. For large-scale decision forest models with 1, 600 trees, netsDB is slightly worse than the lleaves for LightGBM, but it is still slightly better than the Scikit-learn (XGBoost) and ONNX platforms and significantly better than all other CPU platforms. When serving 500 to 1, 600 trees, utilizing GPU significantly accelerated the decision forest prediction. Nvidia FIL and HummingBird with TVM as the backend achieved the best end-to-end performance on GPU.

**Figure 5:**
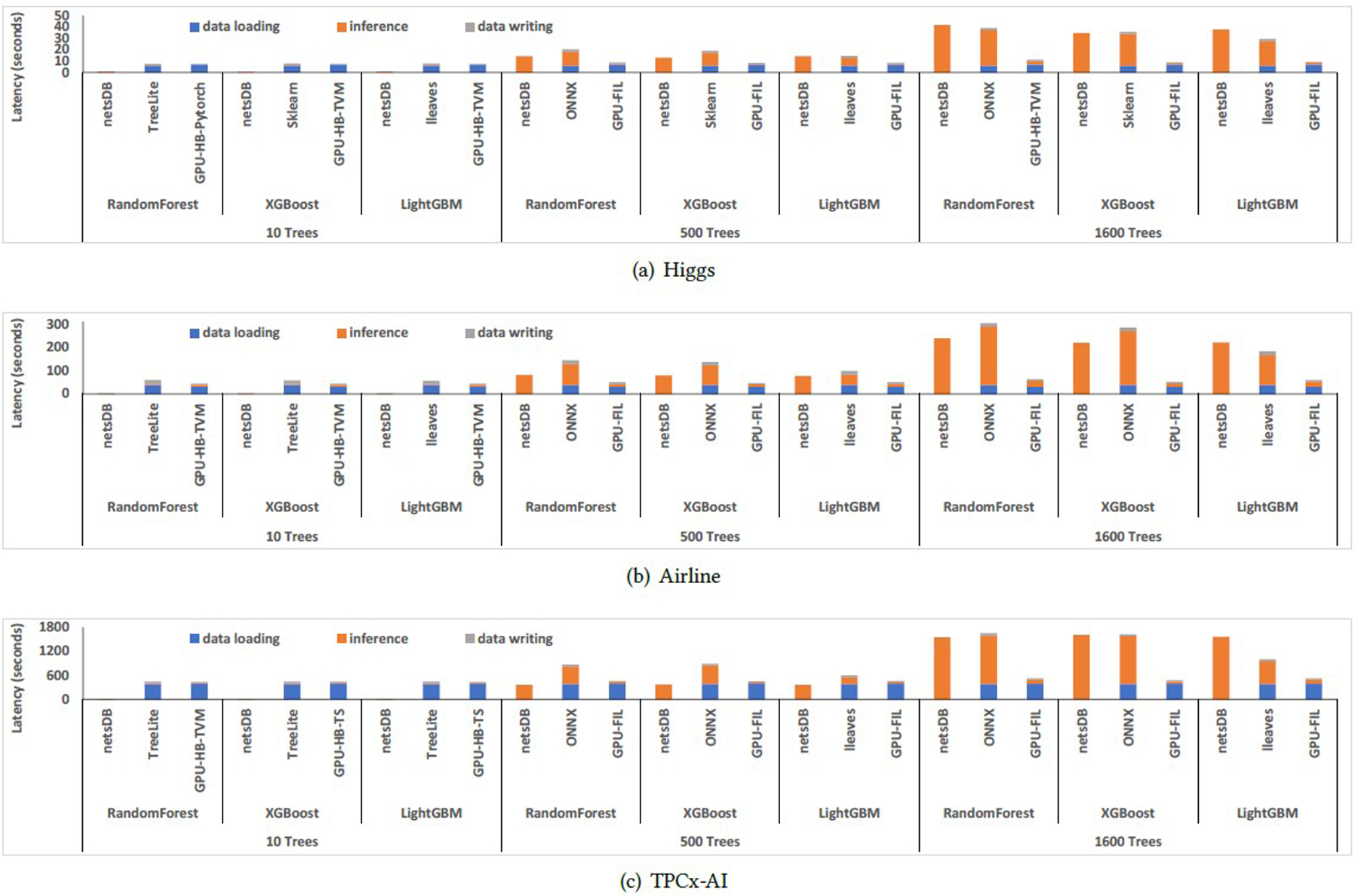
Latency breakdown for the netsDB, the fastest of the rest CPU platforms, and the fastest GPU platform for medium to large datasets.

As illustrated in Fig. 5(a), most of the performance gain when using netsDB was contributed by the avoidance of the data transfer time, which is the major bottleneck for serving small-scale models. However, when the number of trees increases to 1, 600 trees, inference, instead of data transfer, becomes the primary bottleneck, as illustrated in Fig. 5(a). This explains the drop in the performance gain brought by netsDB with the increase in model sizes.

#### 5.4.2 Airline.

As illustrated in Tab. 5, the speedup achieved by netsDB on this workload is even higher than the Higgs workload. In terms of end-to-end time, for 10-tree models, the db-UDF achieved 18×-21× speedup compared to the fastest of the rest of the CPU platforms. For 500-tree models, db-rel achieved 1.3× to 1.9× speedup compared to the fastest of the rest CPU platforms. Similar to Higgs, the performance gain shrinks with the increase in model size. For RandomForest and XGBoost 1, 600-tree models, db-rel achieved 1.3× speedup. For LightGBM with 1, 600 trees, lleaves still performed slightly better than netsDB. In addition, similar to Higgs, the GPU platforms achieved significantly better performance and lower monetary costs than the CPU platforms in most cases.

As illustrated in Tab. 1, the size of the Airline testing dataset is five times larger than Higgs. As a result, the time spent in data transfer accounts for a significantly higher proportion in the end-to-end latency than Higgs, as observed in Fig. 5(b). This explains the increased performance gain of netsDB for the Airline case.

#### 5.4.3. TPCx-AI.

This workload used all 131 million of samples for inferences. With the increase in input dataset size, the data transfer time accounts for an even higher proportion in the overall latency than Higgs and Airline, as illustrated in Fig. 5. Correspondingly, the speedup achieved by netsDB also increased compared to Higgs and Airline, as illustrated in Tab. 6.

Db-UDF achieved more than 100× speedup compared to all other CPU/GPU platforms for the 10-tree models. Moreover, for models with 500 trees, db-rel achieved more than 1.6× to 2.4× speedup compared to the fastest of other CPU platforms, and it achieved 1.2× to 1.3× speedup compared to the fastest of the GPU platforms. Even for 1, 600-tree models, db-rel outperformed all other CPU platforms for RandomForest and XGBoost, though it is slower than lleaves for LightGBM. However, netsDB is slower than most of the GPU platforms in that case due to the increased GPU utilization. Compared to GPU platforms, the CPU platforms can reduce the monetary costs for models with 10 trees and 500 trees by 99% and 44%, respectively. However, GPU can achieve better overall costs for serving models with 1, 600 trees.

### 5.5 Results on Wide and/or Sparse Datasets

We also found that many popular datasets are wide and/or sparse. For example, in Epsilon and Criteo, each tuple has 2000 and 1M features, respectively. Criteo is a sparse dataset that contains missing values. The overall results are illustrated in Tab. 7 and Tab. 8, which are explained as follows.

#### 5.5.1 Epsilon.

As aforementioned, Epsilon is a wide and dense dataset with 2, 000 features. Because PostgreSQL does not support more than 1, 600 columns, we store each sample as one column on PostgreSQL in array type for all platforms except netsDB. In netsDB, the dataset is stored as a collection of tensor blocks in pages, and the page size can be flexibly configured. As illustrated in Tab. 7, netsDB achieved more than 300×, 40×, 10× speedup for models with 10, 500, and 1600 trees, respectively, compared to the fastest of the rest of CPU platforms and all GPU platforms. There are two reasons contributing to such huge performance gains. First, it turns out that it is expensive to convert a PostgreSQL array type back to a NumPy array, which becomes the bottleneck at the inference time in all platforms except netsDB. Second, this dataset contains fewer tuples than other datasets investigated in the paper. Therefore, the inference complexity is significantly lower than other narrower workloads of similar sizes (e.g., Higgs), given the same number of trees and the same depth of each tree.

**Figure 6:**
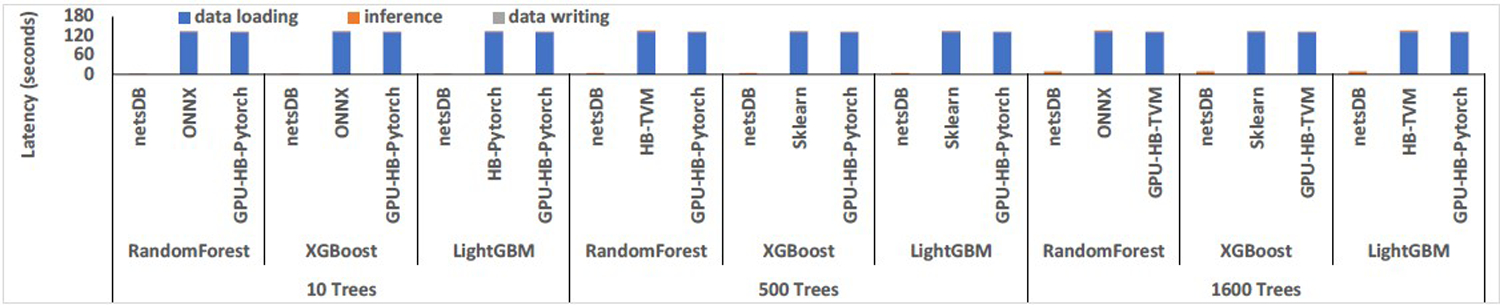
Comparison of Latency breakdown on netsDB, the second best CPU platform, and the best GPU platform for Epsilon

As illustrated in Fig. 6, 99% of time is spent in data loading (including converting the data received from the PostgreSQL array column into a NumPy array). This explains (1) the performance benefits brought by the inference in netsDB compared to other platforms and (2) all other platforms have similar latency (i.e., the data loading overhead, which becomes the bottleneck, is similar for all platforms except netsDB).

#### 5.5.2 Criteo.

Criteo is highly sparse stored in LIBSVM file format [3, 23], where each row contains a row index and a list of <column-index, non-zero-value> pairs, which reduced 80% of storage space. Among the platforms studied in this work, sparse storage formats such as LIBSVM are only supported by Scikit-learn, TreeLite, and netsDB. Therefore, results for other platforms are unavailable. (Using dense format with other platforms failed due to out-of-memory errors.)

According to our observation, although using such a sparse format significantly reduced the end-to-end latency by reducing the data transfer overheads and the memory footprint, however, as a side effect, the ratio of the data transfer latency to the overall latency is also significantly reduced. As a result, less performance gain can be achieved via in-DB inference compared to other workloads that used the dense storage format. As illustrated in Tab. 8, although db-UDF achieved 30× to 60× speedup for 10 trees compared to Sklearn and TreeLite, the performance of db-UDF is significantly worse than TreeLite for 500 and 1, 600 trees.

### 5.6 More Detailed Analysis

**Figure 7:**
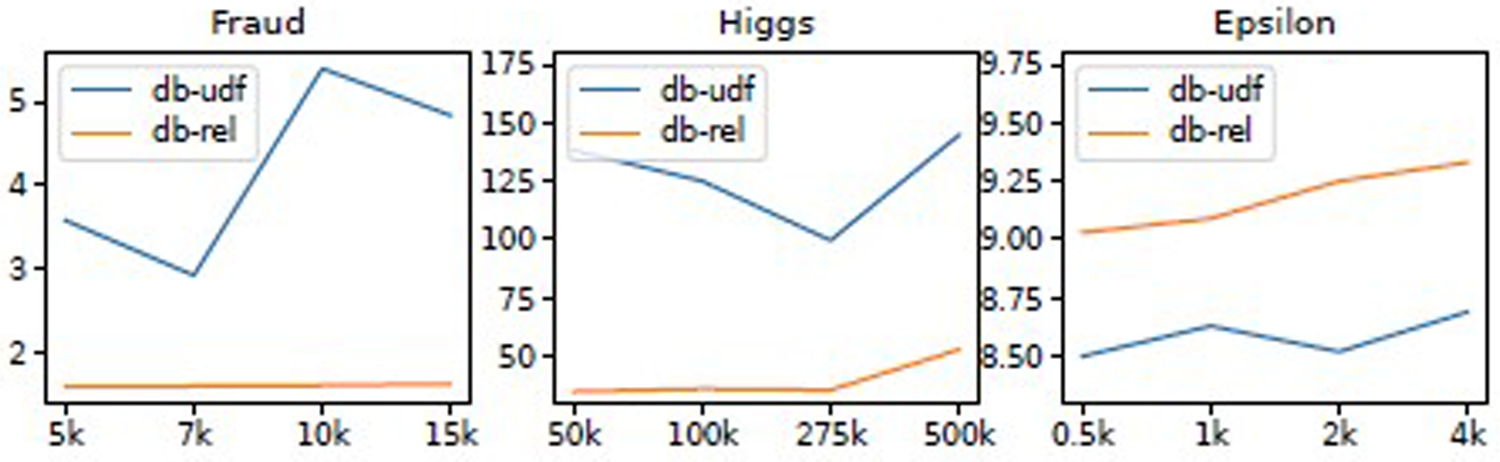
Latency (sec.) vs. block size (bytes) for 1600-tree RandomForest Model

We compare the profiled cache misses and branch misses for UDF-centric and relation-centric representation, as illustrated in Tab. 9. The results are consistent with the latency results for different workloads. In addition, we also analyzed how the tensor block size impacts the latency, and the results are illustrated in Fig. 7. The latency of the UDF-centric implementation is more sensitive to block size than the relation-centric implementation.

### 5.7 Model Conversion

**Figure 8:**
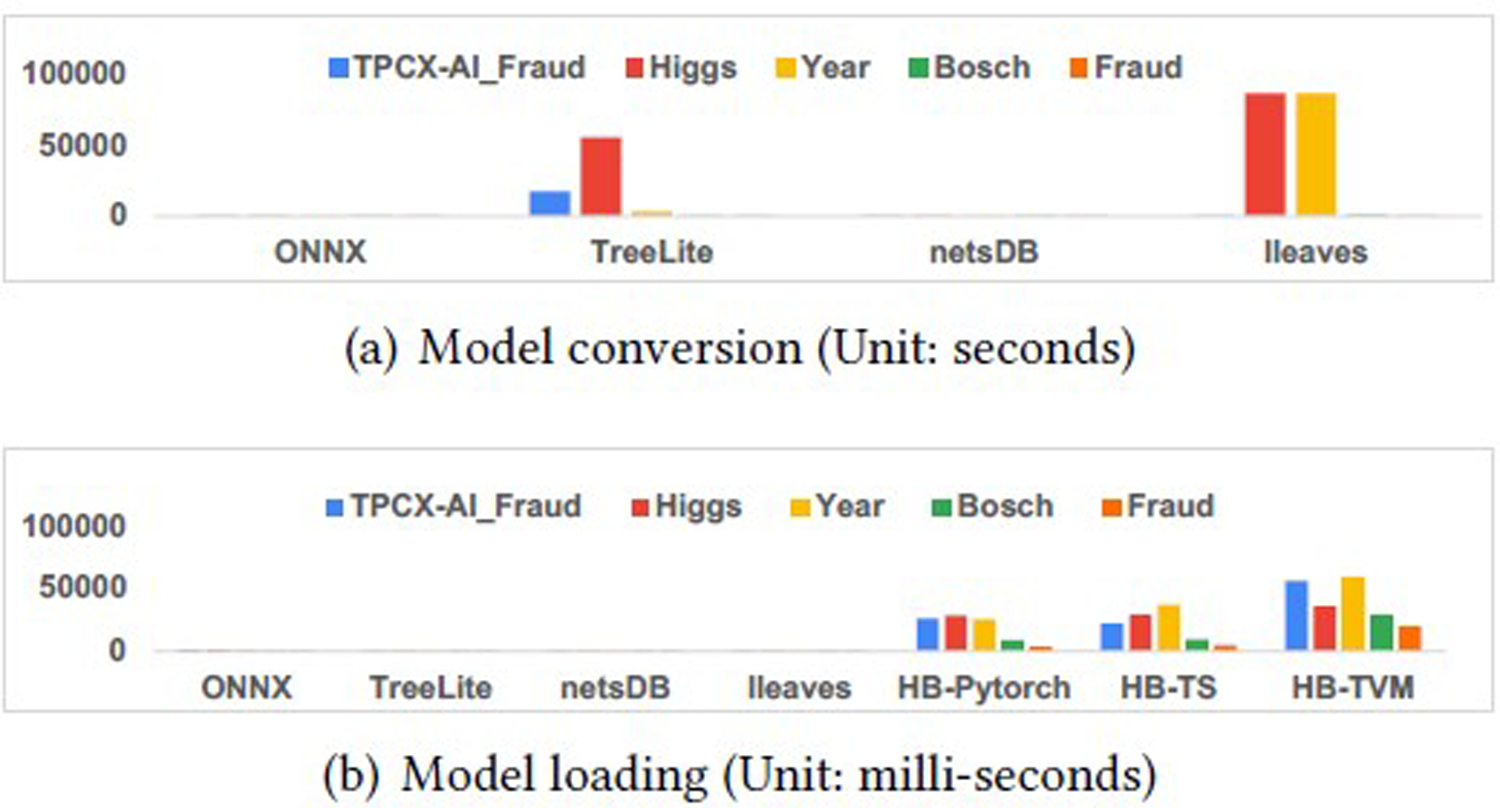
Comparison of model conversion and loading overheads for 1600-tree LightGBM models

As illustrated in Fig. 8(a), the platforms using the compiled tree traversal algorithm, such as TreeLite and lleaves, need more than one day to convert a 1, 600-tree model, which may hinder the adoption of such platforms. The conversion overheads for other platforms are around tens of seconds.

As illustrated in Fig. 8(b), most of the converted models can be loaded in a short time that can be neglected. However, because the converted HummingBird model cannot be persisted, we convert the model during the loading process. That is why HummingBird’s loading process took significantly more time than other platforms.

### 5.8 Validation of DICT Model and Dynamic Representation

**Figure 9:**
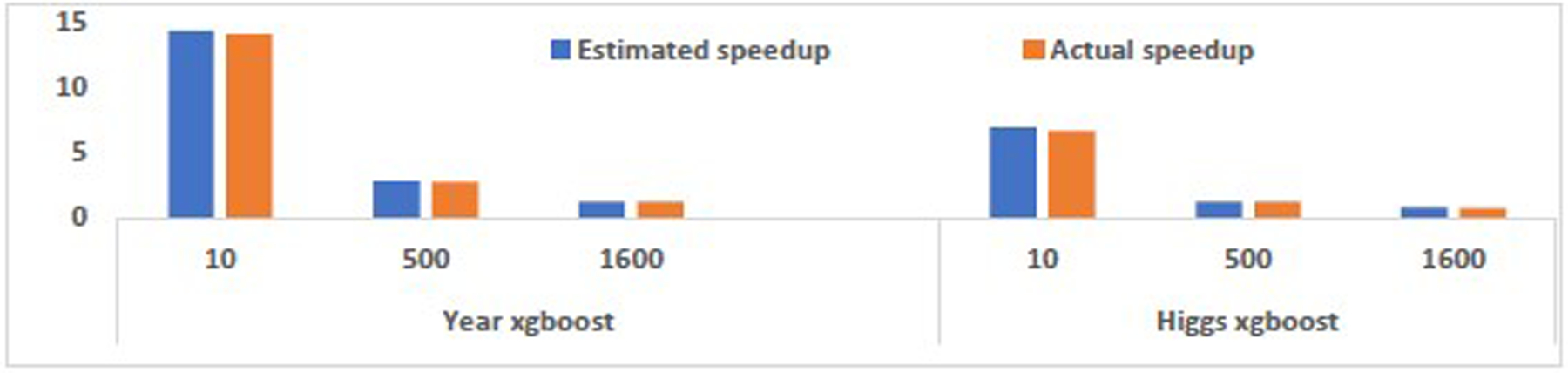
Actual speedup vs. estimated speedup with DICT

This experiment shows that the DICT model accurately predicts the speedup of the in-DB inference over the decoupled inference pipeline. We know the dataset’s size $\left(D\right)$ and cardinality $\left(C\right)$ in the batch inference scenario. The inference throughput is affected by the batch size for some fixed-size models. We measured the inference latency of 100 batches and estimated the inference throughput $\left(I\right)$ by dividing the size of 100 batches of data by the measured latency. Finally, to estimate the data transfer throughput (*T*), we created a dummy dataset that has the same dimensions and data types as the actual dataset and measured the transfer time of the dummy dataset using a high-performance database connector. As illustrated in Fig. 9, the estimated speedups for Year and Higgs datasets are slightly more optimistic but still closely follow the actual speedup.

To validate the idea of rule-based dynamic representation, we trained the decision tree classification model using the inference traces consisting of 1000 inferences of various sizes of models on Fraud, Higgs, Epsilon, and Bosch. We also tested the learned rules extracted from the classification tree on Year, Airline, and TCPx-AI. The learned rules achieved 100% accuracy on the test datasets.

### 5.9 Single-Thread Comparison

TFDF is the only framework that implements the QuickScorer algorithm. However, it does not support multiple threads for inferences, and it incurs significant overheads when invoking the underlying C++-based decision forest library, called Yggdrasil [31], by copying data multiple times. Therefore, we did not consider it in the previous evaluation for fairness. Instead, we compared the performance of XGBoost to various CPU platforms here, all using a single thread. As illustrated in Tab. 10, the results showed that QuickScorer achieved the best inference latency in the single-thread setting. Note that only inference time is measured, and the maximum tree depth is 6 due to the leaf number limit in the QuickScorer implementation [12].

## 6 CONCLUSION

Decision forest, including RandomForest, XGBoost, and LightGBM, is widely used in finance, healthcare, marketing, and search ranking because of its robustness, scalability to large-scale datasets, and model interpretability. However, as shown in this study, most existing benchmarks and evaluations did not consider the management of the inference data, which significantly impacts the overall performance. In this work, we have conducted a comprehensive comparison study for the end-to-end inference performance of decision forest models at different scales on eight platforms. Using various representations, we implemented our own in-database decision forest inference solutions on netsDB.

Our study showed that in-database inferences will save significant data loading/conversion time for workloads where data transfer becomes a significant bottleneck, e.g., inferring large-scale datasets using forest models with tens to hundreds of trees, and small-scale or wide-and-short datasets using all-scale models. Actually, the industry tends to use small decision forest models in production [43], which argues for an in-database inference solution. However, for workloads that exploit large-scale models, where inference rather than data transfer becomes the major performance bottleneck, in-database inference may not be an ideal solution due to its sub-optimal inference performance.

We believe these observations shed some light on the integration of databases and AI/ML and future system design.

## Figures and Tables

**Table 1:** Statistics of the Datasets

Dataset	#Rows	#Features	Prediction Type	Testing Ratio
Epsilon	500K	2000	Classification	20%
Fraud	285K	28	Classification	20%
Year	515K	90	Regression	20%
Higgs	11M	28	Classification	20%
Criteo	51M	1M	Classification	11%
Airline	115M	13	Classification	20%
TPCx-AI	131M	7	Classification	100%

**Table 2:** End-to-End Latency Comparison for Fraud. (Unit: seconds)

	CPU									GPU (latency * GPUCost/CPUCost)		
	Sklearn	ONNX	Hummingbird	TreeLite	lleaves	Spark	db-UDF	db-rel	db-OPT	Hummingbird	FIL	Spark
RandomForest												
10 Trees	1.0	1.0	1.0	1.0	-	2.6	0.5	1.1	**0.3**	**1.3**	1.4	17.9
500 Trees	1.3	1.1	1.5	1.2	-	8.5	1.2	1.2	**0.4**	**1.3**	1.4	18.3
1600 Trees	2.2	1.4	2.8	1.8	-	19.5	2.9	1.6	**0.7**	**1.4**	**1.4**	20.6
XGBoost												
10 Trees	1.0	1.0	1.0	1.0	-	-	0.5	1.1	**0.3**	**1.3**	1.4	-
500 Trees	1.1	1.1	1.7	1.2	-	-	1.0	1.2	**0.4**	**1.3**	1.4	-
1600 Trees	1.3	1.4	2.5	1.4	-	-	1.5	1.5	**0.6**	**1.4**	**1.4**	-
LightGBM												
10 Trees	1.0	1.0	1.0	1.0	1.0	-	0.5	1.0	**0.3**	**1.3**	1.4	-
500 Trees	1.5	1.2	1.7	1.5	1.0	-	1.2	1.3	**0.5**	**1.3**	1.4	-
1600 Trees	2.5	1.6	2.5	2.6	1.2	-	2.5	1.7	**0.8**	**1.3**	1.4	-

**Table 3:** End-to-End Latency Comparison for Year. (Unit: seconds)

	CPU									GPU (latency * GPUCost/CPUCost)		
	Sklearn	ONNX	Hummingbird	TreeLite	lleaves	Spark	db-UDF	db-rel	db-OPT	Hummingbird	FIL	Spark
RandomForest												
10 Trees	4.7	4.7	4.7	4.7	-	5.7	**0.5**	2.3	0.8	**5.4**	5.5	21
500 Trees	5.5	5.2	5.9	6.4	-	17.5	1.4	2.9	**1.3**	**5.5**	5.7	26.4
1600 Trees	7.3	6.5	7.7	10.8	-	43.5	4.8	4.2	**2.6**	**5.7**	**5.7**	37.6
XGBoost												
10 Trees	4.6	4.7	4.7	4.7	-	-	**0.5**	2.3	0.8	**5.4**	5.5	-
500 Trees	5.1	5.1	6.2	6.1	-	-	1.6	2.8	**1.2**	**5.4**	5.5	-
1600 Trees	6.1	5.9	27.1	9.7	-	-	4.8	3.8	**2.3**	**5.7**	**5.7**	-
LightGBM												
10 Trees	4.7	4.7	4.7	4.7	4.7	-	**0.5**	2.3	0.8	**5.4**	5.5	-
500 Trees	6.3	5.2	6.1	6.1	5.0	-	1.5	2.7	**1.2**		5.7	-
										**5.4**		
1600 Trees	10.2	6.1	7.4	9.9	5.9	-	5.0	3.7	**2.2**		5.8	-
										**5.7**		

**Table 4:** End-to-End Latency Comparison for Higgs. (Unit: seconds)

	CPU									GPU (latency * GPUCost/CPUCost)		
	Sklearn	ONNX	Hummingbird	TreeLite	lleaves	Spark	db-UDF	db-rel	db-OPT	Hummingbird	FIL	Spark
RandomForest												
10 Trees	8.3	9.3	8.5	7.8	-	6.7	**0.9**	2.7	1.7	**10.9**	11.9	24.8
500 Trees	28.2	20.3	29.3	42.4	-	129.5	24.4	14.2	**13.1**	13.3	**12.3**	28.3
1600 Trees	70.8	45.2	80.8	130.4	-	556.5	98.6	42.0	**40.2**	**15.5**	15.7	41.6
XGBoost												
10 Trees	8.1	9.3	8.7	8.3	-	-	**0.6**	2.2	1.3	**11.0**	11.3	-
500 Trees	19.1	20.8	33.0	35.3	-	-	25.9	11.6	**10.6**	11.9	**11.7**	-
1600 Trees	35.9	37.6	61.5	99.1	-	-	98.51	31.8	**30.0**	14.3	**12.4**	-
LightGBM												
10 Trees	8.5	9.3	8.8	8.5	8.1	-	**0.9**	2.7	1.8	**11.0**	11.6	-
500 Trees	39.6	18.9	32.8	34.6	14.5	-	26.4	14.2	**13.0**	**12.0**	**12.0**	-
1600 Trees	113.2	39.2	61.1	102.8	**29.5**	-	119	38	36.1	12.9	**12.7**	-

**Table 5:** End-to-End Latency Comparison for Airline. (Unit: seconds)

	CPU									GPU (latency * GPUCost/CPUCost)		
	Sklearn	ONNX	Hummingbird	TreeLite	lleaves	Spark	db-UDF	db-rel	db-OPT	Hummingbird	FIL	Spark
RandomForest												
10 Trees	60.7	74.2	63.5	55.1	-	29.7	**3.3**	17.6	16.2	**64.0**	66.3	68.2
500 Trees	210.6	146.2	273.1	336.3	-	1223.6	302.3	82.4	**80.9**	73.5	**71.1**	94.7
1600 Trees	543.5	305.5	760.8	1052.0	-	5454.4	1120.6	239.4	**236.8**	99.8	**88.3**	149.0
XGBoost												
10 Trees	59.2	73.3	63.8	59.8	-	-	**2.9**	16.7	15.3	**64.0**	65.2	-
500 Trees	143.4	137.8	305.2	264.7	-	-	272.3	80.1	**78.6**	72.5	**67.1**	-
1600 Trees	340.2	87.1	613.1	862.0	-	-	1071.1	220.0	**216.9**	96.7	**73.4**	-
LightGBM												
10 Trees	60.5	74.7	64.3	59.4	58.8	-	**2.8**	18.6	17.3	**64.0**	66.0	-
500 Trees	339.7	144.1	306.4	224.5	98.8	-	96.9	76.5	**74.9**	72.7	**70.4**	-
1600 Trees	1039.2	296.5	614.0	654.8	**185.0**	-	914.8	220.7	218.5	96.8	**84.1**	-

**Table 6:** End-to-End Latency Comparison for TPCx-AI. (Unit: seconds).

	CPU									GPU (latency * GPUCost/CPUCost)		
	Sklearn	ONNX	Hummingbird	TreeLite	lleaves	Spark	db-UDF	db-rel	db-OPT	Hummingbird	FIL	Spark
RandomForest												
10 Trees	449.4	513.3	448.3	450.1	-	116.4	**3.2**	56.2	54.3	625.2	633.5	**314.0**
500 Trees	1233.9	865.1	1635.4	1061.0	-	5396.4	1391.7	363.7	**361.2**	679.5	658.1	**445.8**
1600 Trees	3386.0	1649.3	4311.0	3153.7	-	17299.4	5000.7	1551.4	**1548.2**	856.2	**744.5**	818.8
XGBoost												
10 Trees	438.4	485.5	438.4	441.5	-	-	**2.3**	62.4	58.9	**626.3**	628.5	-
500 Trees	925.7	883.4	1835.4	1560.1	-	-	1328.1	369.9	**368.7**	673.7	**638.8**	-
1600 Trees	2014.7	1614.1	3590.2	4928.4	-	-	4735.7	1612.9	**1610.5**	817.9	**677.9**	-
LightGBM												
10 Trees	445.1	509.9	434.1	434.2	434.	-	**2.8**	63.7	61.2	**625.9**	632.8	-
500 Trees	2069.0	916.7	1876.2	1070.9	586.9	-	1122.0	366.2	**364.7**	676.2	**654.9**	-
1600 Trees	6146.0	1739.3	3646.8	3388.8	**997.2**	-	4623.6	1599.5	1596.7	810.8	**748.5**	-

**Table 7:** End-to-End Latency Comparison for Epsilon. (Unit: seconds)

	CPU									GPU (latency * GPUCost/CPUCost)		
	Sklearn	ONNX	Hummingbird	TreeLite	lleaves	Spark	db-UDF	db-rel	db-OPT	Hummingbird	FIL	Spark
RandomForest												
10 Trees	133.6	132.2	132.5	141.7	-	13.5	**0.7**	6.7	5.7	185.2	186.3	**61.2**
500 Trees	135.5	135.4	135.0	142.4	-	29.5	**2.4**	9.7	7.9	185.5	186.3	**67.6**
1600 Trees	138.1	135.1	135.9	-	-	59.5	**7.9**	12.0	10.9	185.9	186.4	**68.2**
XGBoost												
10 Trees	132.3	132.3	132.4	132.6	-	-	**0.8**	6.7	5.2	185.2	185.9	-
500 Trees	132.6	135.2	135.4	134.6	-	-	**2.4**	8.0	6.4	185.5	186.0	-
1600 Trees	133.1	135.0	137.2	136.6	-	-	**8.5**	11.2	10.7	186.0	186.0	-
LightGBM												
10 Trees	132.7	132.4	132.2	133.0	132.9	-	**0.7**	6.7	5.2	185.2	186.3	-
500 Trees	134.0	134.0	134.3	134.2	135.0	-	**2.4**	8.2	6.9	185.5	186.3	-
1600 Trees	136.2	135.2	135.8	143.1	-	-	**7.9**	12.0	10.9	186.0	186.6	-

**Table 8:** End-to-End Latency for Criteo. (Unit: seconds)

	Sklearn	TreeLite	db-UDF
RandomForest			
Trees	130.8	124.7	**2.2**
500 Trees	409.0	152.1	**79.4**
1600 Trees	1061.7	**216.3**	277.9
XGBoost			
10 Trees	125.2	126.2	**3.99**
500 Trees	209.8	**191.9**	193.1
1600 Trees	412.3	**326.7**	642.2
LightGBM			
10 Trees	132.0	126.6	**4.0**
500 Trees	290.6	**141.7**	172.3
1600 Trees	645.7	**216.2**	564.6

**Table 9:** Profiling for In-Database Inferences (All using XGBoost model with 1600 trees)

	Higgs	Airline	Epsilon	Fraud
Last-Level Cache Misses (Unit: Millions)				
UDF-Centric	2155.1	5271.8	259.5	7.2
Rel-Centric	39.8	313.7	374.7	1.7
Branch Miss Ratio				
UDF-Centric	15.81%	12.21%	15.62%	17.57%
Rel-Centric	0.03%	0.00%	0.25%	5.78%

**Table 10:** XGBoost inference w/ single thread. (Unit: seconds)

#trees	Yggdrasil	TFDF	Sklearn	ONNX	TreeLite	HB-TVM
Higgs						
10	**0.7**	2.1	1.3	1.3	**0.7**	1.9
500	**29.0**	45.4	31.9	34.8	87.6	98.4
1600	**96.5**	140.5	98.9	105.0	340.5	298.1
Fraud						
10	**0.01**	0.31	0.03	0.02	**0.01**	0.03
500	**0.26**	0.63	0.87	0.73	1.34	0.51
1600	**0.48**	1.01	1.67	1.81	3.12	1.44
Year						
10	**0.002**	0.23	0.12	0.06	0.05	0.11
500	1.27	2.86	1.58	1.60	4.08	**0.90**
1600	**4.14**	7.08	4.93	4.87	17.80	2.61

## Summary.

(1) NetsDB outperformed all baselines for small datasets because the data loading process is always a major performance bottleneck (C1 in Sec. 3). (2) Such small datasets are insufficient to fully utilize GPU resources because the gain in inference time cannot compensate for the overheads of transferring data between CPU and GPU (C4 in Sec. 3).

(1) On the CPU platforms, the in-DB inference on netsDB outperformed other platforms in most cases, except that it is slightly worse than lleaves for large-scale model sizes (e.g., 1, 600 trees). That is because, when model size increases, the data loading process becomes a less severe performance bottleneck.

The inference overheads could scale significantly faster than the data transfer overheads when the dataset fits the OS buffer cache (C1/C2 in Sec. 3). (2) GPU platforms outperformed netsDB on TPCx-AI, Higgs, and Airline using 500-tree and 1600-tree models. However, netsDB significantly outperformed all GPU platforms for 10-tree models, which is a more popular use case according to a recent industrial study [43] (C5 in Sec. 3). (3) Db-rel significantly improved the performance of decision forest models that have 500 and 1600 trees, compared to the db-UDF, by reducing cache misses, and even branch misses (See Tab. 9).

(1) The in-DB inferences represented by netsDB outperformed all baselines for the wide Epsilon dataset stored in dense format. That is because the data loading is more of a performance bottleneck for dense datasets that are *short-and-wide* than *tall-and-narrow* datasets of similar sizes (C3 in Sec. 3). (2) GPU is less helpful for the inferences over short and wide datasets than tall and narrow datasets of similar sizes. That is because the short and wide datasets contain fewer testing samples, reducing the workload’s computational complexity. (3) If a large dataset is extremely sparse (e.g., Criteo), a sparse storage format will significantly reduce the storage costs and the performance benefits of in-DB inferences will diminish correspondingly (C1/C4 in Sec. 3).

## CCS CONCEPTS

- Computer systems organization
- Computing methodologies → Machine learning
